# Primary Malignant Mesothelioma of the Tunica Vaginalis Presenting as a Benign Scrotal Mass: A Case Report

**DOI:** 10.7759/cureus.90608

**Published:** 2025-08-20

**Authors:** Zihni Can Dagdeviren, Onur Amac, Yelda Dere, Engin Derekoylu

**Affiliations:** 1 Urology, Faculty of Medicine, Mugla Sitki Kocman University, Mugla, TUR; 2 Pathology and Laboratory Medicine, Mugla Sitki Kocman University, Mugla, TUR

**Keywords:** hemi scrotum, hydrocele, malignant mesothelioma, tunica vaginalis tumor, urology and oncology

## Abstract

Primary malignant mesothelioma of the tunica vaginalis is an exceptionally uncommon neoplasm, comprising a very small fraction of all mesothelioma cases. Due to its nonspecific clinical presentation, it is frequently misdiagnosed as benign scrotal conditions, such as a hydrocele. In our case, a 77-year-old male presented with a gradually enlarging swelling in the left hemiscrotum. Scrotal Doppler ultrasonography (USG) and magnetic resonance imaging (MRI) revealed a hydrocele with polypoid masses and nodular soft tissue components of the tunica vaginalis. Tumor markers were normal. Surgical exploration revealed multiple solid masses on the testis and spermatic cord. Histopathologic examination confirmed malignant mesothelioma of epithelioid type, supported by immunohistochemical reactivity for calretinin, cytokeratin 7 (CK7), cytokeratin 5/6 (CK5/6), pan-cytokeratin (PanCK), Wilms tumor 1 (WT1), and loss of BRCA1-associated protein-1 (BAP1). Following the diagnosis of primary tunica vaginalis mesothelioma, the patient underwent radical inguinal orchiectomy and hemiscrotectomy. Adjuvant chemotherapy with pemetrexed and cisplatin was administered. No recurrence was detected after seven cycles, and the patient remains disease-free. This case highlights the diagnostic challenges of tunica vaginalis mesothelioma because of its infrequency and clinical similarity with benign processes. Diagnosis needs to be based on multimodal imaging, histopathology, and immunohistochemistry. Early and total surgical intervention followed by adjuvant therapy may hold the key to successful treatment.

## Introduction

Mesothelioma is a malignant tumor of mesenchymal origin that originates from mesothelial cells lining serous cavities such as the pleura, peritoneum, pericardium, and scrotum. Mesothelioma of the tunica vaginalis originates from the mesothelium lining the outer surface of the tunica albuginea and the inner layer of the scrotum [1]. Primary involvement of the tunica vaginalis is extremely rare, and only a limited number of cases have been reported in the literature. The incidence of this entity accounts for less than one percent of all pleural and peritoneal mesotheliomas [1,2]. The first recorded case of tunica vaginalis mesothelioma was reported by Barbera and Rubino in 1957, and fewer than 300 cases have been reported to date [2,3]. Four percent of cases are bilateral [2]. Clinically, it commonly presents as a hydrocele, but palpable mass and inguinal pain may also be present [4]. It can also be detected incidentally. Although it usually occurs in the sixth and seventh decades of life, it can even be seen under the age of 20 [1,5]. Although asbestos exposure is generally involved in the etiology, asbestos exposure has been detected in 30-40% of reported tunica vaginalis mesotheliomas [1,2]. There is no standard protocol for diagnosis and treatment approaches. In this report, we aim to draw attention to this rare disease by presenting the clinical, radiological, and pathological findings and treatment process of a patient diagnosed with primary tunica vaginalis mesothelioma. 

## Case presentation

**Figure 1:**
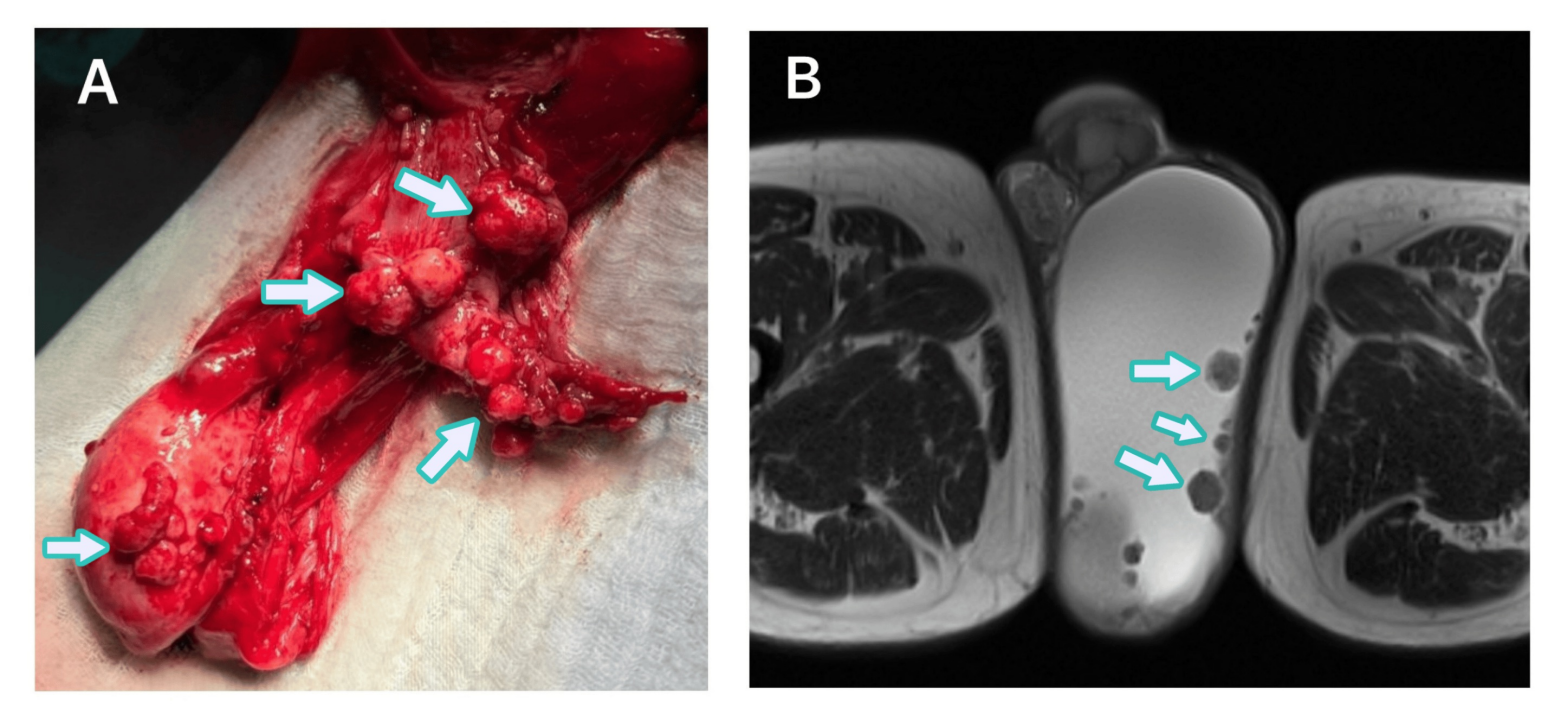
Scrotal exploration and scrotal magnetic resonance imaging (MRI). (A) Scrotal exploration revealed solid tumoral lesions arising from the surface of the testis and spermatic cord. Arrows show tumoral solid lesions. (B) Scrotal MRI revealed solid tumoral lesions located on the tunica vaginalis, in association with a hydrocele and the adjacent testis. Arrows show tumoral solid lesions.

A male patient aged 77 presented to our urology clinic in July 2024 with a complaint of gradually increasing left scrotal swelling over the past six months. Physical examination revealed an approximately 10-cm, fluctuant, painless mass in the left scrotum. The right testis was normal. The patient was retired from a desk job and had no history of asbestos exposure. Scrotal Doppler ultrasonography revealed bilateral intense hydrocele, predominantly on the left, reaching 8 cm, with exophytic extensions in the left testicular parenchyma and isoechoic polypoid lesions up to 8 mm. MRI showed a 12-cm cystic area with nodular soft tissue intensities at the level of the left testis (Figure 1B). Tumor markers (beta-human chorionic gonadotropin (β-hCG), alpha-fetoprotein (AFP), and lactate dehydrogenase (LDH)) were within normal limits (Table 1). In a surgical exploration in October 2024, multiple solid suspicious lesions with a diameter of 1-2 cm were observed on the testis and spermatic cord (Figure 1A).

**Table 1:** Laboratory results of the patient.

Test name	Patient value	Reference range	Interpretation
Hemoglobin (Hb)	13.8 g/dL	13.5-17.5 g/dL	Normal
White blood cell (WBC)	7.84 × 10⁹/L	4.0-10.0 × 10⁹/L	Normal
Platelet count (PLT)	296 × 10⁹/L	150-400 × 10⁹/L	Normal
Lactate dehydrogenase (LDH)	157 U/L	135-225 U/L	Normal
β-human chorionic gonadotropin (β-hCG)	<0.200 IU/L	0-5 IU/L	Normal
Alpha-fetoprotein (AFP)	1.57 ng/mL	0-10 ng/mL	Normal
Urea	27 mg/dL	10-50 mg/dL	Normal
Creatinine	0.74 mg/dL	0.6-1.3 mg/dL	Normal
C-reactive protein (CRP)	4.5 mg/L	0-5 mg/L	Normal

**Figure 2:**
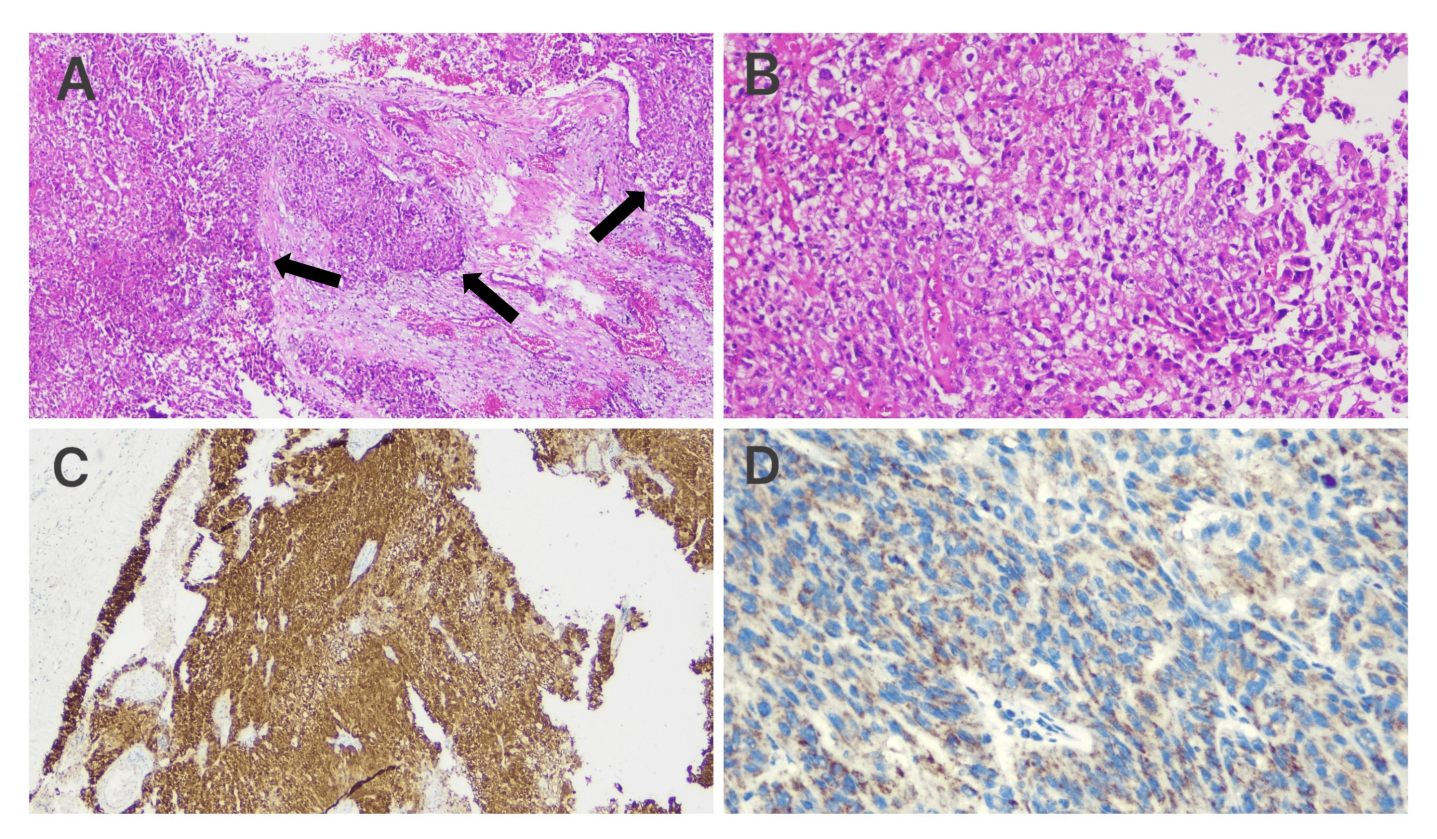
Microscopic examination images. (A) Solid epithelial tumoral nests of mesothelioma, 40x, hematoxylin and eosin (H&E). Arrows show malignant mesothelial cell proliferations. (B) Solid epithelial tumoral nests of mesothelioma, 100x, H&E. (C) Diffuse calretinin positivity, 40x, Diaminobenzidine (DAB). (D) Loss of nuclear staining with BAP1, 100x, DAB. BAP1, BRCA1-associated protein 1

The lesions were excised, and hydrocelectomy was performed (Figure 1A). Macroscopic examination of the pathological specimen revealed a gray-cream-colored parietal layer of tunica vaginalis measuring 9 x 8 x 2.8 cm in total, with gray-yellow nodular lesions ranging from 2.5 x 1.5 x 1 cm to 0.2 cm in diameter on its surface. Microscopic examination revealed an infiltrative tumor with solid nests, a focally epithelioid appearance, pleomorphic, hyperchromatic nuclei, and prominent mitotic figures distributed within the desmoplastic stroma (Figures 2A-2B).

Immunohistochemical staining showed diffuse (+) staining for calretinin, patchy (+) staining for CK7, (+) staining for CK5/6, (+) staining for PanCK, (+) staining for WT1, (-) staining for CK20, (-) staining for GATA3, (-) staining for TTF-1, (-) staining for S100, (-) staining for p40, (-) staining for PAX8, (-) staining for p63, (-) staining for NKX3.1, (-) staining for CDX2, (-) staining for SALL4, (-) staining for CD30 (Table 2), and loss of nuclear staining with BAP1 (Figures 2C, 2D). The patient was diagnosed with mesothelioma based on morphological and immunohistochemical findings. Subsequent postoperative radiological screenings to investigate the origin revealed no other focus or metastasis. The case was considered Primary Tunica Vaginalis Mesothelioma. Subsequently, the patient underwent left radical inguinal orchiectomy and hemiscrotectomy in December 2024. Pathological examination revealed no residual lesions in the testicular parenchyma and epididymis, both macroscopically and microscopically. The patient was started on adjuvant chemotherapy with cisplatin and pemetrexed. Control computed tomography scans after seven cycles of chemotherapy showed no residue or recurrence. The patient completed chemotherapy seven months after diagnosis and continues to live disease-free.

**Table 2:** Immunohistochemical profile of the tumor.

Marker (Abbreviation)	Result
Calretinin	Diffuse (+)
Cytokeratin 7 (CK7)	Patchy (+)
Cytokeratin 5/6 (CK5/6)	(+)
Pan-cytokeratin (PanCK)	(+)
Wilms tumor 1 (WT1)	(+)
Cytokeratin 20 (CK20)	(-)
GATA binding protein 3 (GATA3)	(-)
Thyroid transcription factor-1 (TTF-1)	(-)
S100 protein (S100)	(-)
p40	(-)
Paired box gene 8 (PAX8)	(-)
p63	(-)
NK3 homeobox 1 (NKX3.1)	(-)
Caudal type homeobox 2 (CDX2)	(-)
Spalt-like transcription factor 4 (SALL4)	(-)
Cluster of differentiation 30 (CD30)	(-)
BRCA1-associated protein 1 (BAP1)	Loss of nuclear staining

## Discussion

The tunica vaginalis develops from the protrusion of the peritoneum during the embryonic development period and therefore contains a mesothelial layer, from which mesothelioma can develop [6,7]. The prevalence in the literature is reported as 0.2 per million [8]. Although its etiology is not clear, as with other mesotheliomas, publications are reporting that asbestos exposure may play a role, and this etiology is seen in 30%-40% of cases [2]. In addition, previous trauma, previous inguinal hernia surgery, radiation to the scrotal area, long-term hydrocele, and previous epididymitis have also been considered as potential etiological factors [9]. It is mostly seen in the sixth and seventh decades of life, but cases have also been reported in childhood and young adulthood in the literature [10]. Clinical and radiological findings may mimic more common conditions such as hydrocele, hernia, epididymitis, or other scrotal tumors. Careful differential diagnosis and further investigations are therefore important. Scrotal Doppler USG and MRI play an important role in the evaluation of scrotal masses, but they may not always be sufficient to differentiate mesothelioma from other scrotal pathologies. As in our case, the definitive diagnosis is usually made by histopathological examination after surgical exploration. Macroscopically, it can present as thickening of the tunica vaginalis, multiple nodular or papillary lesions within the hydrocele sac, and solid nodular lesions surrounding the testicular parenchyma. Our case, like most cases in the literature, presented as nodular formations within the hydrocele sac. As with other mesotheliomas, tunica vaginalis mesothelioma has three subtypes: epithelioid, sarcomatoid, and biphasic (1). The most common histological subtype is the epithelioid subtype, as in our case [2,11]. Although it mostly exhibits papillary and tubular structures, it can also be seen as solid nests [4,12]. In our case, solid nests are more predominantly observed. Immunohistochemically, positivity for Calretinin, CK7, CK5/6, WT1, thrombomodulin, epithelial membrane antigen (EMA), podoplanin, epithelial cell adhesion molecule antibody(BerEp4), and negativity for CK20 and CEA are helpful in the diagnosis [2,11]. In addition, loss of nuclear expression of BAP1 supports the diagnosis [13,14]. In our case, diffuse (+) staining for Calretinin, patchy (+) staining for CK7, (+) staining for CK5/6, (+) staining for PanCK, (+) staining for WT1, (-) staining for CK20, and loss of BAP1 nuclear expression were detected. GATA3, TTF-1, S100, p40, PAX8, p63, NKX3.1, CDX2, SALL4, CD30 (-) were detected to rule out metastatic disease. There is no standard protocol for diagnosis and treatment approaches. Surgical excision is the primary treatment method for primary tunica vaginalis mesothelioma [15]. Radical orchiectomy and hemiscrotectomy ensure complete removal of the tumor. In advanced stages or high-risk patients, adjuvant chemotherapy and radiotherapy options can be evaluated. In this case, adjuvant chemotherapy was administered with the recommendation of oncology, considering the high risk. The prognosis of primary tunica vaginalis mesothelioma depends on the stage of the tumor, histological type, and completeness of surgical resection. In this case, the tumor being limited to the tunica vaginalis and the absence of residual tumor may indicate a good prognosis. However, long-term follow-up and monitoring for recurrence are important for the patient. In the literature, there are a limited number of case reports on this tumor, and most of these cases presented as complicated hydrocele or scrotal mass. Diagnosis was usually made by surgical exploration and histopathological examination, and treatment included surgical excision and adjuvant chemotherapy. This case presents a rare presentation of primary tunica vaginalis mesothelioma, highlighting the challenges in the diagnostic process and the importance of multimodal diagnostic methods. Furthermore, it demonstrates that a multidisciplinary treatment approach is critical in the management of this rare disease. Further research on primary tunica vaginalis mesothelioma is important, especially for the standardization of diagnostic and treatment approaches and the determination of prognostic factors. Molecular pathology and genetic analyses may contribute to a better understanding of this disease and the development of targeted therapies. 

## Conclusions

Malignant tunica vaginalis mesothelioma is a rare tumor that may be misdiagnosed as benign scrotal conditions such as hydrocele, rendering diagnosis challenging. In the present case, with no evidence of metastasis at presentation, the diagnosis was confirmed through histopathological and immunohistochemical analyses, demonstrating loss of BAP1. The patient underwent early radical surgery followed by chemotherapy with pemetrexed and cisplatin. No recurrence was observed after completion of seven treatment cycles. This case highlights the importance of keeping in mind the differential diagnosis of unusual swellings in the scrotum, including mesothelioma, and considering the benefits of early active treatment.
